# Willingness to Engage in Collective Action After the Police Killing of an Unarmed Black Man: Differential Pathways for Black and White Individuals

**DOI:** 10.1002/ajcp.12587

**Published:** 2022-02-15

**Authors:** Brynn E. Sheehan, Valerian J. Derlega, Ralitsa S. Maduro, Delaram A. Totonchi

**Affiliations:** ^1^ Department of Psychiatry and Behavioral Sciences Eastern Virginia Medical School, Healthcare Analytics and Delivery Science Institute Norfolk Virginia USA; ^2^ Psychology Department Old Dominion University Norfolk Virginia USA; ^3^ Sentara Healthcare, Quality Research Institute Virginia Beach Virginia USA; ^4^ Center for Advanced Study of Teaching and Learning University of Virginia Charlottesville Virginia USA

**Keywords:** collective action, defensive motivational system (DMS), multigroup invariance testing, path analysis, police violence, race

## Abstract

This cross‐sectional survey study examined the underlying psychosocial constructs of Black (*n* = 163) and White (*n* = 246) university students' willingness to endorse racially motivated collective action. Consistent with the defensive motivation system model, we expected the police shooting of an unarmed Black American to activate concerns about personal safety, thereby eliciting negative affect, lack of forgiveness of the perpetrator, and motivation to engage in collective action. This path model was expected for both Black and White participants, with stronger associations among Black participants. In the full model, Black participants identified more with the victim and indicated greater personal threat, which led to (1) more negative affect and greater endorsement of collective action and (2) greater avoidance of the shooter and greater endorsement of collective action. In the Black participants model, collective action was explained by identifying with the victim and feeling personally threatened. In the White participants model, collective action was explained by three pathways stemming from identifying with the victim and personal threat, including negative affect, seeking avoidance, and seeking revenge. The results indicate different mechanisms to explain Black and White individuals' motivation to endorse collective action to prevent police‐involved shootings of unarmed Black Americans.

## Highlights

- Differences between Black and White individuals' motivation of collective action for racial justice.
- For Black individuals, action was explained by identifying with the victim and feeling threatened.
- For White individuals, action was explained by negative affect and lack of forgiveness.

## INTRODUCTION

The high‐profile killing of unarmed Black Americans by White police officers (including, among others, Oscar Grant, Michael Brown, Tamir Rice, Walter Scott, Deravis Caine Rogers, and recently, Breonna Taylor, Andre Hill, and George Floyd) has generated enormous outrage about the use of lethal and excessive force in police–citizen encounters with people of color (Lowery, 2016; McCarthy, 2020). Many Americans, including elected officials, have denounced these killings of Black citizens. For instance, after the killing of Mr. Floyd, Jacob Frey, the mayor of Minneapolis, said, “Being Black in America should not be a death sentence” (Stockman, 2020). Street protests, demonstrations, and riots erupted in response to the police brutality, both protesting these incidents and calling attention to the broader problem of racial injustice manifested by police killings of Black Americans.

Individuals of all races have participated in these protests (Nimtz, 2020; Shepherd & Hauser, 2016). Nevertheless, research indicates that Black, compared to White, Americans are more willing to support these protests and similar acts of collective action (see Pew Research Center, 2016; Thomas & Horowitz, 2020). The current study examined the underlying constructs that motivate Black and White individuals to become involved in collective action to prevent the race‐related killings of Black Americans by police. We assume that indirect exposure to police shootings of Black Americans (via social media and/or traditional news sources) activates concerns, especially for Black Americans, about being a possible target of police brutality (Schwartz & Jahn, 2020). Further, when faced with a status quo deemed to be unjust, White Americans, too, may join in solidarity with the victim and/or minority group to challenge authority and promote change (Subašić et al., 2008). We expect that these concerns activate a defensive motivational system (DMS) that, according to Pietrzak et al. (2005), “facilitates the monitoring and detection of threat‐relevant cues and prepares the individual for swift responses when cues of danger are detected” (p. 69). The awareness of one's vulnerability to being harmed should lead to strong reactions (emotional and behavioral) to prevent the threat from happening. Based on the DMS model, we examined, among Black and White individuals, how identification with the shooting victim, concern for one's own safety, the amount of distress (negative affect) related to the shooting incident, and unwillingness to forgive the shooter (represented by avoidance and seeking revenge) predict collective action.

## MECHANISMS LINKING RACE AND INVOLVEMENT IN COLLECTIVE ACTION

### Identifying with the victim and feeling personally threatened

Identification with a particular group (i.e., one's social identity) is especially salient for individuals who perceive that their group has been collectively disadvantaged and/or treated poorly (Sellers et al., 1998; Tajfel & Turner, 1979). For example, Black Americans, as members of a historically disadvantaged group in US society, are much more likely than Whites to see their race as central to their identity (74% of Black vs. 15% of White Americans say their race/ethnicity is important to how they see themselves; Horowitz et al., 2019). Social identity theorists argue that when individuals identify as members of a traditionally oppressed group, incidents of injustice that happen to other members of the group are seen as “self‐relevant” and are likely to motivate action to redress these grievances (Becker & Tausch, 2015). Identifying with individuals of a systematically oppressed group has been noted as the driver for collective action among many civil rights leaders. As the late US Representative John Lewis shared in his last essay on social justice reform and activism, “I will never ever forget the moment when it became so clear that he [Emmet Till, a 14‐year‐old Black adolescent who was murdered in Mississippi in 1955] could easily have been me” (Lewis, 2020).

Based on social identity theory, when a Black person is the victim of a violent incident (as in a police‐involved shooting of an unarmed Black man), we would expect Black individuals to identify with the victim due to the saliency of race as it relates to their self and social identity (Becker & Tausch, 2015). Although racial group identity, and therefore an identification with the victim, is likely elicited more strongly among Black individuals, research exploring models of social change have highlighted the overreliance and misunderstandings of the in‐group/out‐group relations (Reynolds et al., 2015; Subašić et al., 2008). Subašić et al. (2008) notes, according to the political solidarity model, that intergroup dynamics are needed to achieve social and political change. Specifically, White individuals, acting in solidarity with the minority to challenge an authority that is seen as unjust, may redefine their self‐categorization to align or identify with the minority/victim and view the authority as the out‐group. When considered in concert with social identity theory and the DMS model, these theories help elucidate a pathway of collective action. That is, the political solidarity and social identity theories support why individuals would identify with victims or minority groups that are treated unjustly, and once this identification occurs, the DMS model explains that a sense of heightened threat should then motivate action. It is therefore expected that while Black individuals may more strongly identify with the Black victim of a police‐involved shooting, experience a greater sense of personal threat, and be more willing to engage in collective action, White individuals, too, may identify with the victim, report feelings of threat, and indicate intentions to participate in collective action for social and political change.

### Negative affect as a response to personal threat

Research has documented the relationship between personal experiences with racial discrimination and poor mental health (Bor et al., 2018; Carter et al., 2017). Identifying with victims of a violent crime and feeling threatened can be considered secondary victimization and is related to a myriad of negative emotional responses. For example, a recent study focusing on the nine church congregant victims killed by a white supremacist in Charleston, South Carolina, and Tamir Rice killed by a police officer in Cleveland, Ohio, found that Black Americans who identified strongly with their racial group were more likely to identify with these Black victims, felt more personally threatened by the shootings, and experienced greater negative affect (Roberts et al., 2017). Both *identification with the victim* and *feeling threatened* were associated with greater anxiety, depression, and anger/hostility. Based on the DMS model, we expect that Black, compared to White, Americans are more likely to identify with Black victims of police‐involved shootings and, in turn, feel more personally threatened by these incidents, which should be associated with greater endorsement of emotional and behavioral responses to the threat.

Of note, much of the literature regarding emotions and action focuses on anger as a predictor of action (e.g., Gill & Matheson, 2006; Mackie et al., 2000; van Zomeren et al., 2012). Argued to be an emotion‐focused means of coping with injustice, anger has been found to mediate the association between perceptions of injustice and endorsement of collective action (van Zomeren et al., 2004, 2012). Anger has also been used to explain support for the Black Lives Matter movement and willingness to engage in collective action to fight racial injustice (Selvanathan et al., 2018), to protest hate crimes committed against Black and lesbian, gay, and bisexual students on a college campus (Mallett et al., 2008), and to support political action on behalf of Iraqi citizens after the Second Gulf War (Pagano & Huo, 2007).

We add to this discussion that feeling personally threatened is likely to elicit several negative emotions, including anger, depression, and anxiety, that is, a combination of negative emotions that should motivate collective action for change. Although depression (Holahan et al., 2005) and anxiety (Borkovec et al., 2004) are traditionally associated with avoidance as a coping mechanism, research has identified a positive relationship between negative affect and collective action (Perez‐Garin et al., 2016). We suggest that these emotional responses, that is, anger, reflecting outrage about the threat posed by the police‐involved shooting; depression about the long‐term nature of the threat; and anxiety about the danger posed, prompt collective action. We expect these relationships to be particularly strong among Black Americans who, consistent with social and self‐identity theory, are likely to more strongly identify with Black shooting victims and subsequently feel personally threatened by the event compared to White Americans (Sellers et al., 1998; Tajfel & Turner, 1979). Based on the DMS, the greater emotional response should indicate a greater propensity to act in such a way as to protect oneself from current and future risks. We expect this to be evidenced by the endorsement of collective action.

### Lack of forgiveness toward the shooter

Offering forgiveness to the shooter(s) may also be a response to the unjustified killing of Black Americans (Rodriguez, 2019). There is a concern, especially among some Black American leaders and Black opinion writers, that forgiveness of racial discrimination and violence targeting African Americans might suppress motivation to combat racism (see Anderson, 2018; Hawes, 2019, p. 92). We focus on two components of lack of forgiveness that might incentivize collective action: motivation for avoidance and motivation for revenge. Avoidance concerns evading, for instance, stimuli, thoughts, and emotions related to the shooter or perpetrator, while revenge focuses on wanting to get even or wanting the perpetrator to pay for their actions (McCullough et al., 1998). Both the motivations for avoidance and revenge, consistent with the DMS model, focus on personal protective behaviors designed to maintain a social distance from the police shooter and seek retribution for the harm done to the shooting victim. As such, endorsement of either reaction (avoidance or revenge) are expected responses to the shooting and may be associated with collective action if the individual believes that engaging in such collective effort could protect themselves from threat in the future.

## STUDY PREDICTIONS

Based on our prior theorizing, we expected that Black individuals (for whom a Black social identity should be salient), as compared to White individuals, would identify more with the shooting victim, perceive a greater threat to self, more negative affect, less forgiveness for the shooter, and more strongly endorse collective action. Based on the DMS model, we also expected that similar pathways would explain White individuals' endorsement of collective action. White Americans, as members of an advantaged group, may see less police‐directed violence in mostly White communities. Nevertheless, White Americans who see Black and White persons as belonging to a “superordinate category” that includes individuals from disadvantaged and advantaged groups, and who endorse fairness for all (see Gaertner & Dovidio, 2000; Subašić & Reynolds, 2009; Subašić et al., 2008), may redefine their self‐categorization and view others (anyone who is indifferent or not critical of the injustice perpetrated by the police‐involved killing of unarmed Black victims) as the out‐group. These individuals would be expected to identify with Black victims and view the killing of a Black man as a threat to self (based on the recategorization of self as belonging to an “ingroup” made up of anyone who might feel vulnerable about potentially unfair treatment by the police). Thus, White individuals who identify more with the shooting victim should be more likely to endorse collective action via the mediating pathways of personal threat, negative affect, and lack of forgiveness for the shooter.

## METHOD

### Participants

Research participants were 163 (39.9%) Black and 246 (60.1%) White university students who were enrolled at a public university in southeastern Virginia. The majority of participants were female (64.81%), with a mean age of 26.93 years (SD = 10.00). Participants were freshmen (16.39%), sophomores (13.01%), juniors (21.69%), seniors (28.67%), graduate students (18.80%), and “other” (1.45%). The majority of participants (67.64%) reported a family income between $30,001 and $120,000 and reported that their mothers (78.45%) and fathers (73.18%) had either completed high school, some college, or had completed college.

### Procedure

The study was announced via email to White and Black students who were identified via demographic information provided by the Office of Student Activities. Interested participants consented to complete an anonymous, online survey, providing their reactions to recent police‐involved shootings in the United States. After providing electronic consent, participants completed demographic and neuroticism measures. Next, participants read the account of the fatal shooting of Mr. Crutcher. We used the following vignette, based on news sources available shortly after the incident, to describe the shooting (Carissimo, 2016; Stack, 2016):

On September 16th, 2016, police in Tulsa, Oklahoma responded to several 911 calls reporting that a vehicle was stopped in the middle of a highway. Upon arriving at the scene, the officers found Terence Crutcher, a 40‐year‐old African American man, walking around his car with his hands raised. As the officers approached Mr. Crutcher, he reportedly began to reach into his pockets and into his car. Suspecting he was reaching for a gun, an officer tasered and shot Mr. Crutcher. Mr. Crutcher was pronounced dead at a local hospital later that day. Upon searching Mr. Crutcher's belongings and car, police found PCP in Mr. Crutcher's car, but they did not find a firearm.^1^

After reading the vignette, participants completed several measures about their reactions to the fatal shooting. At the completion of the survey, participants were given an opportunity to leave comments and had the option to be redirected to another website where they were entered into a raffle for one of four $50 Amazon gift cards. At the end of the survey, participants were reminded that their responses were anonymous and were provided with the email address of one of the investigators if they sought more information about the study.

### Predictor, mediator, and outcome variables

#### Demographic and predictor variables

Demographic information was obtained on participant's age, year in school, an estimate of family income, mother's and father's highest level of education, and race. The primary exogenous variable was the race of participants (Black or White).

#### Mediating variables

Three of the mediators, *Identification with the Victim, Personal Threat, and Lack of Forgiveness*, were rated on a five‐point Likert scale from 1 (*Strongly disagree*) to 5 (*Strongly agree*). See Appendix A for a complete list of the scales that were constructed for this study (identification with the victim, personal threat, and the outcome variable of collective action).

##### Identification with the Victim

We used a four‐item scale measuring the degree to which participants identified with the victim, modified to reflect Mr. Crutcher. The scale has shown to be valid and reliable in previous research assessing violence against LGBTQ individuals (Maduro et al., 2020). Sample items include: “I could put myself in the shoes of Mr. Crutcher”; and “I feel connected with Mr. Crutcher.” Reliability for identification with the victim measure was *α* = .91.

##### Personal Threat

We used a four‐item scale measuring how vulnerable participants felt based on what happened to the shooting victim, modified to specify Mr. Crutcher. The feelings of personal threat scale have previously been shown to be valid and reliable in research assessing identification with and violence against LGBTQ (Sheehan et al., 2020). Sample items include: “I feel personally threatened by the police officers' shooting of Mr. Crutcher”; and “I feel like what happened to Mr. Crutcher could happen to me.” Reliability for the measure was *α* = .96.

##### Negative Affect

Participants completed the Multiple Affect Adjective Check List‐Revised (Lubin & Zuckerman, 1999) to indicate how they felt emotionally about the vignette. Participants checked all the words that described their current feelings, providing scores on measures of negative affect (10 items for anxiety, 12 items for depression, and 14 items for anger/hostility). Example anxiety items include: “afraid,” “fearful,” “nervous”; example depression items include: “discouraged,” “miserable,” “suffering”; and example anger/hostility items include: “disgusted,” “angry,” “enraged.” Summated scores were used to operationalize each of the affect subscales. Consistent with previous literature, a composite score of negative affect was created (Henson et al., 2013). Subscale reliabilities were: *α* = .82 for anxiety, *α* = .81 for depression, and *α* = .83 for anger/hostility. The reliability for the composite measure of negative affect was *α* = .92.

##### Lack of Forgiveness

The Transgression‐Related Interpersonal Motivations Inventory was administered to assess Avoidance and Revenge related to the perpetrator (McCullough et al., 1998). Participants responded to 12 items assessing their thoughts and feelings toward the police officers involved in the shooting of Mr. Crutcher. Sample items of Avoidance include: “I would find it difficult to act warmly toward them” and “I live as if they don't exist or aren't around.” The reliability for Avoidance motivation was *α* = .94. Sample items of Revenge include: “I want them to get what they deserve” and “I wish that something bad would happen to them.” The reliability for Revenge motivation was *α* = .82.

#### Outcome variable

##### Collective Action

We constructed a six‐item measure of willingness to engage in collective action as a response to the shooting. The statements were adapted, in part, from items used by the Pew Research Center (2016) to measure Black and White Americans' attitudes about community engagement to address questions of racial equality. The items presuppose the injustice committed by the officer's killing of Mr. Crutcher and focus on nonviolent ways of creating change in police work, including political action and peaceful demonstrations (Becker & Tausch, 2015; Hasan‐Aslih et al., 2019). Sample items include: “I would like to participate in a march or rally to keep things like the police shooting of Mr. Crutcher from happening again”; and “I would donate to charities such as the Institute for Community Police Relations or the NAACP Legal Defense Fund to help prevent things like the police shooting of Mr. Crutcher from happening again.” Responses were made on a five‐point Likert scale from 1 (*Strongly disagree*) to 5 (*Strongly agree*). Mean scores were used to operationalize the measure. Reliability was *α* = .94.

## RESULTS

### Preliminary analyses

Before conducting analyses, data were examined for normality. No extreme outliers were found and kurtosis and skewness values were found to be within the normal range for all variables. Descriptive statistics and bivariate correlations by race can be found in Table 1. Of note, mean scores on all critical variables in the study were higher among Black compared to White participants, including identifying with the victim, personal threat, negative affect, avoidance, revenge, and collective action. The path model was tested with 5000 bootstrap replications (Mooney & Duval, 1993; Shrout & Bolger, 2002) via the full information maximum likelihood method to maximize the use of all available data. All analyses were conducted using Mplus Version 8 (Muthén & Muthén, 2011).

**Table 1:** Listwise bivariate correlations and descriptive statistics for Black (*n* = 163) and White (*n* = 246) participants

Variable	1	2	3	4	5	6	7	8	9	10	11
1. Age	–	**−0.03**	**0.16**	**0.06**	**0.06**	**−0.07**	**−0.07**	**0.001**	**−0.02**	**0.01**	**−0.09**
2. Gender	−0.01	–	**0.14**	**−0.03**	**0.04**	**−0.15**	**−0.11**	**−0.26** **	**−0.08**	**0.01**	**−0.25** **
3. Family's income	−0.14	0.03	–	**0.31** **	**0.33** **	**−0.05**	**−0.09**	**−0.09**	**−0.03**	**−0.21** *	**−0.17**
4. M Education	−0.24**	0.09	0.18*	–	**0.49** **	**0.05**	**0.10**	**−0.02**	**−0.02**	**−0.05**	**−0.01**
5. F Education	−0.31**	0.14	0.25**	0.50**	–	**−0.11**	**−0.06**	**−0.10**	**0.01**	**−0.15**	**−.13**
6. ID with Victim	−0.10	−0.11	0.01	0.13	0.10	–	**0.53** **	**0.38** **	**0.09**	**0.29** **	**0.45** **
7. Personal Threat	−0.10	−0.05	0.01	0.09	0.11	0.60**	–	**0.50** **	**0.44** **	**0.37** **	**0.55** **
8. Negative Affect	−0.04	−0.24**	0.08	0.04	−0.02	0.44**	0.44**	–	**0.30** **	**0.25** **	**0.43** **
9. Avoidance	−0.14	−0.06	−0.06	0.12	0.21**	0.56**	0.65**	0.49**	–	**0.43** **	**0.24** **
10. Revenge	−0.13	−0.07	−0.01	0.10	0.10	38**	0.48**	0.41**	0.59**	–	**0.18** *
11. Collective Action	−0.15*	−0.15*	−0.06	0.12	0.07	0.63**	0.55**	0.57**	0.67**	0.54**	–
M_Black_	25.51	0.28	2.60	3.60	2.98	3.70	3.87	10.27	3.72	2.57	4.12
M_White_	28.26	0.38	3.17	3.45	3.40	1.95	1.77	6.24	2.51	1.60	2.68
SD_Black_	9.25	–	1.38	1.36	1.44	0.99	1.10	7.87	0.89	0.94	0.82
SD_White_	10.53	–	1.48	1.39	1.62	0.93	0.93	6.21	1.33	0.69	1.22

*Note*: Values in bold on the top diagonal represent correlations for Black participants and values on the bottom diagonal represent correlations for White participants. M Education = mother's education; F Education = father's education; ID with Victim = identification with victim. Gender was coded as 1 = female, 2 = male. Family's income was coded as 1 = below $30,000; 2 = $30,001–$60,000; 3 = $60,001–$90,000; 4 = $90,001–$120,000; 5 = $120,001–$150,000; and 6 = $150,001 and above. Parent's education was coded as 1 = some high school; 2 = completed high school; 3 = some college; 4 = completed college; 5 = some graduate school; and 6 = completed graduate school. Race was coded as 0 = White, 1 = Black.

* *p* < .05

** *p* < .001.

### Full sample model with race as the exogenous variable

**Figure 1:**
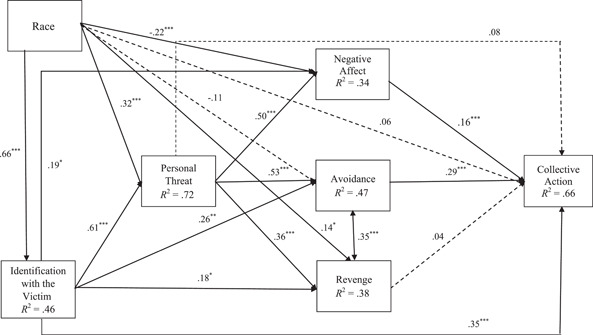
Full sample model predicting collective action with race as the exogenous variable. *Note*: Model with standardized path coefficients. Significance is based on bootstrapped confidence interval (CI). Dashed paths are not significant; solid paths are significant at *p < .05, **p < .01, and ***p < .001, and 95% CI does not include 0. Model effects are adjusted for gender. Race is coded as 0 = White, 1 = Black

The overall model was found to have excellent fit, χ^2^ (5) = 15.24, *p* = .010, CFI = 0.99, TLI = 0.97, RMSEA = 0.07, and SRMR = 0.02. Path estimates and significance of direct effects can be found in Figure 1. The indirect path coefficient estimates with bootstrapped standard errors and confidence intervals (CIs) are represented in Table 2. In addition to multiple direct effects, two sequential mediational effects were found, including the relationship between race and collective action via identifying with the victim, feeling personally threatened, and experiencing negative affect (*b* = 0.03, SE_b_ = 0.01, 95% CI [0.02, 0.05]), as well as identifying with the victim, feeling personally threatened, and seeking to avoid the perpetrator (*b* = 0.06, SE_b_ = 0.02, 95% CI [0.03, 0.09]). Consistent with our hypotheses, these significant indirect paths indicated that (a) Black, compared to White, participants identified more with the victim, indicated greater personal threat, more negative affect, and greater endorsement of collective action; and (b) Black, compared to White, participants identified more with the victim, indicated greater personal threat, greater motivation to avoid the shooter, and greater endorsement of collective action. The variance explained in each variable is presented in Table 4.

**Table 2:** Indirect path estimates with bootstrapped SEs and CIs for the full sample model: identification with the victim as a primary mediator

Path	*b*	SE	*β*	95% CI
Race → Identification with the Victim → Collective Action	**0.60**	**0.11**	**.23**	**[0.39, 0.82]**
Race → Identification with the Victim → Negative Affect → Collective Action	**0.05**	**0.02**	**.02**	**[0.004, 0.10]**
Race → Identification with the Victim → Avoidance → Collective Action	**0.13**	**0.05**	**.05**	**[0.04, 0.22]**
Race → Identification with the Victim → Revenge → Collective Action	0.01	0.01	.01	[−0.02, 0.04]
Race → Identification with the Victim → Personal Threat → Collective Action	0.08	0.07	.03	[−0.06, 0.22]
Race → Identification with the Victim → Personal Threat → Negative Affect →Collective Action	**0.08**	**0.02**	**.03**	**[0.04, 0.13]**
Race → Identification with the Victim → Personal Threat → Avoidance → Collective Action	**0.16**	**0.04**	**.06**	**[0.08, 0.25]**
Race → Identification with the Victim → Personal Threat → Revenge → Collective Action	0.02	0.02	.01	[−0.02, 0.05]

*Note*: SEs and confidence intervals (CIs) are based on unstandardized estimates. Significant indirect paths are presented in bold. Significance was inferred based on the bootstrapped 95% CIs. Indirect paths were marked as significant if the CIs did not contain zero.

### Multigroup invariance testing

**Figure 2:**
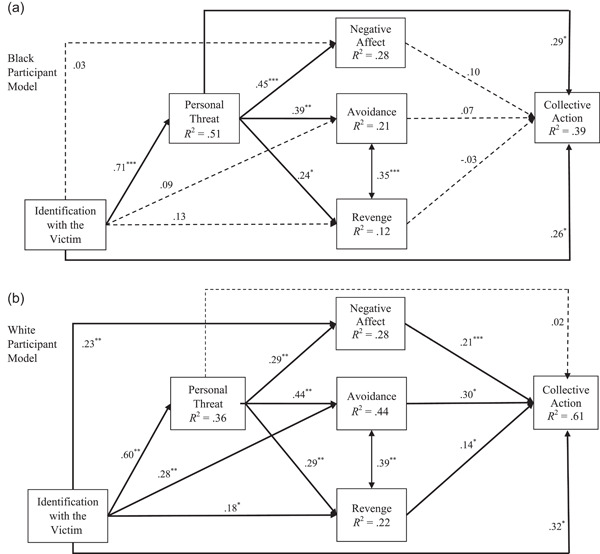
Race‐based models predicting collective action. *Note*: Black and White models with standardized path coefficients. Dashed paths are not significant; solid paths are significant at *p < .05, **p < .01, and ***p < .001, and 95% confidence interval (CI) does not include 0. Model effects are adjusted for gender

Results of the multigroup invariance testing revealed that, consistent with our hypotheses, the overall path model significantly differed for White versus Black participants, *χ* ^2^ (19) = 87.369, *p* < .001. See Figure 2 for path estimates and significance of direct effects for each model by race. Indirect path estimates with bootstrapped standard errors and CIs can be found in Table 3. See below for path model details by racial group.

**Table 3:** Indirect path estimates with bootstrapped SEs and CIs for the model tested separately by race: identification with the victim as a primary mediator

	Black				White			
Path	*b*	SE	*β*	95% CI	*b*	SE	*β*	95% CI
Identification with the Victim → Personal Threat → Collective Action	**0.18**	**0.08**	**.21**	**[0.02, 0.33]**	0.02	0.05	.01	[−0.07, 0.11]
Identification with the Victim → Personal Threat → Negative Affect → Collective Action	0.03	0.02	.03	[−0.02, 0.07]	**0.05**	**0.02**	**.04**	**[0.01, 0.08]**
Identification with the Victim → Personal Threat → Avoidance → Collective Action	0.02	0.02	.02	[−0.03, 0.06]	**0.11**	**0.03**	**.08**	**[0.05, 0.18]**
Identification with the Victim → Personal Threat → Revenge → Collective Action	−0.01	0.01	−.01	[−0.03, 0.02]	**0.04**	**0.02**	**.03**	**[0.004, 0.07]**

*Note*: SEs and confidence intervals (CIs) are based on unstandardized estimates. Significant indirect paths are presented in bold. Significance was inferred based on the bootstrapped 95% CIs. Indirect paths were marked as significant if the CIs did not contain zero.

#### Black participants' model

When stratified to examine the path model for Black respondents only, results indicated that identifying with the victim (*b* = 0.26, SE_b_ = 0.11, 95% CI [0.05, 0.48]) and feelings of personal threat (*b* = 0.29, SE_b_ = 0.13, 95% CI [0.02, 0.42]) were significantly associated with collective action. Greater identification with the victim was strongly associated with greater personal threat (*b* =0.71, SE_b_ = 0.05, 95% CI [0.62, 0.80]), and more threat was associated with increased negative affect (*b* = 0.45, SE_b_ = 0.09, 95% CI [0.28, 0.62]), seeking avoidance (*b* = 0.39, SE_b_ = 0.13, 95% CI [0.14, 0.63]), and seeking revenge (*b* = 0.24, SE_b_ = 0.11, 95% CI [0.02, 0.46]). These results are consistent with our hypotheses. The only indirect relationship to emerge was between identification with the victim and collective action via personal threat (*b* = 0.21, SE_b_ = 0.09, 95% CI [0.03, 0.38]). Thus, greater identification with the victim was associated with greater concern of personal threat, which was then associated with greater endorsement of collective action (see Figure 2a). See Table 4 for the variance explained in each variable.

**Table 4:** Variance explained in study variables separated by model

	*R* ^2^ in each model		
Variables	Full	Black participants	White participants
Identification with the Victim	46%	10%	1%
Personal Threat	72%	51%	36%
Negative Affect	34%	28%	28%
Avoidance	47%	21%	44%
Revenge	38%	12%	22%
Collective Action	66%	39%	61%

*Note*: The full sample model included Race as an exogenous variable and adjusted for gender, which explains the variance explained in Identification with the Victim, whereas the race‐based models only adjusted for gender.

#### White participants' model

Results indicated that identifying with the victim (*b* = 0.32, SE_b_ = 0.05, 95% CI [0.21, 0.42]), experiencing negative affect (*b* = 0.21, SE_b_ = 0.05, 95% CI [0.12, 0.30]), seeking revenge (*b* = 0.14, SE_b_ = 0.05, 95% CI [0.04, 0.25]), and seeking avoidance (*b* = 0.31, SE_b_ = 0.07, 95% CI [0.16, 0.45]) were significantly associated with collective action among White respondents. Greater identification with the victim was associated with greater personal threat (*b* = 0.60, SE_b_ = 0.05, 95% CI [0.51, 0.69]), negative affect (*b* = 0.22, SE_b_ = 0.08, 95% CI [0.07, 0.37]), revenge (*b* = 0.17, SE_b_ = 0.07, 95% CI [0.03, 0.32]), and avoidance (*b* = 0.27, SE_b_ = 0.07, 95% CI [0.13, 0.41]). Indirect paths were found between identifying with the victim and collective action via (1) personal threat and negative affect (*b* = 0.04, SE_b_ = 0.01, 95% CI [0.01, 0.06]), (2) personal threat and avoidance (*b* = 0.09, SE_b_ = 0.03, 95% CI [0.04, 0.14]), and (3) personal threat and revenge (*b* = 0.03, SE_b_ = 0.01, 95% CI [0.003, 0.06]). Specifically, these indirect paths indicated that: (a) White participants' greater identification with the victim was associated with higher personal threat, more negative affect and greater collective action; (b) greater identification with the victim was associated with higher personal threat, more negative affect, greater avoidance, and more collective action; and (c) greater identification was associated with higher personal threat, more negative affect, greater revenge, and more collective action (see Figure 2b). The variance explained in each variable is presented in Table 4.

## DISCUSSION

The current research documents underlying mechanisms that help explain why Black and White Americans are motivated to support collective action in the aftermath of a Black man being killed by police. Stratified models highlighted key differences between Black and White respondents that motivate action. In our full sample model, Black, compared to White, Americans more strongly identified with the shooting victim (Mr. Crutcher), which, in turn, was associated with greater concern for one's own safety. This concern by Black Americans is warranted given that a recent model predicted that 1 in 1000 Black men and boys will be killed by police (Edwards et al., 2019). This initial path supports the theory of self‐identity and the extension of social identity discussed previously (Sellers et al., 1998; Tajfel & Turner, 1986). Given the long history of racially motivated injustice in the United States, Black individuals' self‐ and social‐identity are likely elicited when incidences, such as the shooting of Mr. Crutcher, occur as the incident is part of a larger collective schema of discrimination and racial bias by the police. Meaningful differences, in terms of the most salient paths, were found when the full model was explored separately by the race of the participant.

### Comparing Black versus White participants' willingness to endorse collective action

The full model documents why Black Americans, as members of a group that has been the frequent target of racial injustice in the United States, may be more likely than White Americans to support and/or engage in collective action in the aftermath of the police killing of an unarmed Black person. Black, compared to White, participants identified more with the shooting victim and indicated greater concerns about the personal threat. In turn, more concerns about the personal threat were associated with more negative affect, greater motivation to avoid the shooter, and greater collective action intentions. These results are consistent with ideas from social identity theories (Sellers et al., 1998; Tajfel & Turner, 1979) that violence directed at a member of a victimized group will heighten group identification for other members of the group. Our work was able to quantify the size of that direct relationship and show that race predicted 46% of why a person may identify with the victim (see Table 4). Indeed, consistent with study predictions Black compared to White participants identified more with the shooting victim and perceived themselves as less safe after the shooting.

The results also support the defensive motivation system model (Pietrzak et al., 2005), which argues that the perception of threat should motivate thoughts and feelings that facilitate strategies to reduce the risk of harm now and in the future. Consistent with the DMS model, study findings indicate a positive association between perception of threat, negative affect, and collective action intentions. Also, a greater threat was associated with greater avoidance of the shooter and greater collective action intentions. Greater avoidance of the shooter could reflect an awareness that the police shooter's behavior was unjustified and that the current state of policing—especially officers' treatment and use of force in encounters with Black persons—requires correction (see Desilver et al., 2020). It is worth noting that revenge, as a component of lack of forgiveness, did not play a role in the full sample model.

#### Black Americans' willingness to endorse collective action

When examining the model for Black participants, a primary path appeared. Among the Black participants, those who identified more with the shooting victim were more concerned about being threatened personally by the incident, which was associated with greater endorsement of collective action. Given that many Black Americans share the view that Blacks are treated unfairly in dealing with the police and that they may have been stopped unjustifiably by the police themselves (Desilver et al., 2020), it is understandable that Black participants who highly identify with the shooting victim are more willing to endorse collective action via concerns about the personal threat. We had initially expected that negative affect and lack of forgiveness (avoidance and revenge) would be proximal predictors of collective action among the Black participants. Nevertheless, it appears that identification and perception of threat are enough to activate the DMS, motivating collective action to eliminate the threat of police violence. Further, the role of threat as a mediator is in line with the results of other studies documenting how the perception of threat to members of one's social group mobilizes collective action. For instance, Barreto et al. (2009) documented how Latinos protested in the Spring of 2006 in more than 160 cities against a House Bill (Bill 4437) that would have imposed penalties on undocumented immigrants and employers who hired them. The researchers argued that the Latino community felt personally threatened by the proposed policy, which, in turn, motivated political activism by members of the community. Current study results for Black participants provide further support for this view that identifying with a victim elicits concern for one's own safety, thereby motivating action against the threat‐inducing act.

Although identifying with the victim and feeling personally threatened were the only two variables that predicted collective action for Black participants, individuals who perceived their own safety to be at risk after reading about the incident experienced greater distress (i.e., anxiety, depression, anger/hostility) and increased feelings of avoidance and revenge. Previous research supports the relationship between racial discrimination, secondary victimization of racially motivated violence, and poor mental health (Bor et al., 2018; Roberts et al., 2017). However, previous research has also reported that anger is an emotional coping response to a stressor, and elicits collective action (Mallett et al., 2008; van Zomeren et al., 2012). Nevertheless, in the current results predicting who among Black Americans endorsed collective action, negative affect was not a proximal predictor of collective action.

#### White Americans' willingness to participate in collective action

The White participant model did not reveal a single indirect effect of identification with the victim to collective action through personal threat, and instead highlighted three sequential mediational paths that stemmed from identification with the victim and personal threat to action: negative affect, avoidance, and revenge. These results indicate that, for the White participants, identifying with the shooting victim and concerns about personal threat are not sufficient to explain their willingness to endorse collective action. First, the model showed that White participants who identified more with the shooting victim reported greater concern about the personal threat, which then predicted greater negative affect and greater endorsement of collective action. This finding, at least among the White participant sample, indicates how activation of the DMS can heighten negative affect that can stimulate collective action. The finding also extends previous research on the role of emotions predicting collective action, which has focused primarily on anger. Study results support that a combination of emotions, anxiety, depression, and anger, serve as emotive responses in the aftermath of these events, and contribute to the motivation of collective action, at least among respondents who identified as White.

There were two additional paths that, for White participants, mediated the relationship between identifying with the victim, feeling personally threatened, and indicating an intention to engage in collective action; the motivation to avoid the shooter and the motivation to seek revenge. Identifying with the shooting victim and concerns about personal threat may activate intermediate strategies (avoidance and revenge) to mitigate police violence. Avoidance may reflect an unwillingness not only to interact with the police shooter but an unwillingness to interact with other police officers who may be seen as posing a threat of violence. Revenge, on the other hand, likely reflects a motive for the shooter (and the police generally) to make amends for the unjust shooting of Mr. Crutcher. The relationship between lack of forgiveness and collective action found among the White participant model may indicate that the motivation to engage in action is representative of the motivation to address the broader issue of racially motivated injustice and police brutality and not an act against the individual perpetrator or officer.

#### Comparing race‐based mechanisms for collective action

In explaining how mechanisms for collective action may differ for Black and White Americans, identifying with the victim and feeling threatened may mean different things for Black and White individuals. Given that Black Americans are especially concerned about unfair treatment by the police and are generally more knowledgeable about race‐based police–citizen encounters (Desilver et al., 2020), the Black participants in our study may have more easily imagined themselves in the position of the Black shooting victim and felt less safe. White Americans, as members of a group that is less likely to have violent encounters with the police (Desilver et al., 2020; Schwartz & Jahn, 2020) may have a more abstract view of what it means to identify with the minority group and feel threatened in the aftermath of a fatal shooting of a Black person. Thus, the White participants may have required additional mechanisms (including negative affect and lack of forgiveness) to motivate collective action.

The constructs investigated in the current study can be directly tied to the racial events of 2020, both in terms of the racially motivated violence that occurred and the collective action that resulted. Following the 2020 deaths of Ahmaud Arbery, Breonna Taylor, and George Floyd, US citizens responded online and on the streets, demanding justice for the victims and expressing outrage against the racial injustices in the country. Three days after George Floyd's death, there were 8.8 million uses of the #BlackLivesMatter (Pew Research Center, 2020). By summer 2020, an estimated 15–26 million people participated in over 10,000 protests across the United States, making it one of the largest movements in the country's history (Buchanan et al., 2020). Findings from the current study would suggest that in response to seeing, hearing, and reading about these murders, individuals likely experienced a range of psychological responses, including perceptions of threat to their own safety (Black participants) and negative affect and lack of forgiveness (White participants), which helped motivate participation in these online and in‐person acts of protest. Although Black individuals are more likely to have identified with the victim(s) and felt personally threatened, which predict their collective action, White respondents also experience feelings of negative affect and lack of forgiveness, which predict their likelihood of participation.

### Limitations

The results are useful in explaining what underlying cognitive constructs motivate Black and White individuals to endorse collective action in the aftermath of a police killing of an unarmed Black individual. Nevertheless, several limitations of the research should be noted. First, we urge caution in extrapolating results as data are cross‐sectional and do not reflect causal relationships. It is possible, for instance, that endorsing collective action may increase an individual's identification with victims and concerns about being a victim of police violence if one participated in a demonstration with police presence. Second, we relied on Black and White American university students as research participants. This reliance on young adults is justified given that men and women between the ages of 20 and 30 years, regardless of race, are at the greatest risk of being killed by police (Edwards et al., 2019). Our research also focused on group responses; however, it would be worthwhile examining how the centrality of one's identity (see Sellers et al., 1998) affects intentions to engage in collective action via the sequential mediators that we have examined. As individuals of various backgrounds have supported efforts to improve racial injustice (Fisher, 2020), future research would benefit from extending this study to other racial, ethnic, and/or religious minority groups that may be disproportionately targeted by the police (e.g., Latinx, Asian/Pacific Islanders, Muslims).

It should be noted that the tested models explained a considerable amount of variance in the mediational and endogenous constructs; however, the White model explained substantially more variance in collective action (60%) compared to that explained in the Black model (39%). This difference may be due to other constructs we did not account for in our model that are salient for Black individuals, such as personal experiences with prejudice and racial discrimination (Sheehan et al., 2019) and centrality of racial identity (Sellers et al., 1998). It is also possible that the psychological literature in general, which has historically relied on White participants, may be biased in its construct development, validation, and theory testing. Researchers should be cognizant of the samples used when developing and validating psychological measures and the impact that homogeneous samples have on generalizability across racial and other minority groups.

We should also note that the current research was conducted in the Fall 2016 semester, shortly after the killing of Mr. Crutcher by a police officer. Since then, there have been numerous deaths and injuries of Black American men and women by the police (Statista, 2021), including graphic videos documenting these disturbing police encounters (e.g., New York Times, 2018). Despite the date of our data collection, data remain relevant and findings from the current study directly align with the collective action seen in 2020.

### Practical implications

Social activists, that is, persons working to achieve political or social change, who are combating racial bias in police–citizen encounters, should consider the value of the DSM model (Pietrzak et al., 2005) in understanding what might motivate individuals' willingness to engage in collective action. The police shooting of Mr. Crutcher (and of other Black Americans who were killed in race‐involved police shootings) increases people's collective action intentions, particularly when individuals can put themselves in the shoes of the victim and understand the racialized aspects of these events. Thus, key features for social activists are to provide important details of the event in the broader context of racial injustice and acknowledge and humanize the victim. If and when individuals can identify with the victim, they too experience a sense of threat. For White individuals, this threat is also related to heightened negative affect and lack of forgiveness for the shooter, which are necessary to promote action.

It is also worth sharing information with law enforcement, community leaders, and mental health professionals that racialized police shootings can have an adverse effect on mental health, particularly within minority communities. Supporting this view, a recent study by Bor et al. (2018, June 21) examined the association between the number of police killings of unarmed Black Americans within a state 3 months before a survey of self‐reported mental health problems (defined as the number of days in the previous month when the respondent reported that her/his mental health was “not good”). Findings indicated that the number of killings of unarmed Black Americans in the state was associated with a greater number of poor mental health days for Black (but not for White) respondents living in the same state. The killing of unarmed White Americans or armed Black Americans in the state was not associated with mental health problems for either Black or White participants living in the same state. Based on our results and the DMS model, it is likely that the police killings of unarmed Black Americans increase identification with the shooting victim and concerns about the threat to self and significant others (including family, friends, and the greater Black community). When such killings happen, and Black individuals feel powerless to remediate what is perceived as a racialized pattern of police killings of unarmed Black Americans, mental health problems will likely occur.

### Conclusions

In the aftermath of police killings of unarmed Black Americans in the United States, persons of various races across the globe have come together to protest against police brutality directed against Black Americans. Given the impact of protests on social change and the widespread support for these protests and demonstrations, understanding the psychological pathways that underlie individuals' decision to protest is important. Our study revealed clear differences between Black and White individuals in the motivation to engage in collective action to prevent the police killing of unarmed Black persons in the future. Specifically, among Black participants, collective action was largely motivated by identifying with the victim and experiencing the associated feelings of personal threat. Alternatively, White participants were motivated by a series of other responses stemming from identifying with the victim and feeling threatened, including experiencing negative affect and a lack of forgiveness toward the shooter. These results highlight the importance of resisting a single explanation to account for who is participating in collective action and why. Future research should examine how various contexts affect mechanisms for Black and White activism in the face of racial injustice.

## CONFLICT OF INTERESTS

The authors declare that there are no conflict of interests.

## Footnotes

1 Although not contained in the vignette read by the participants, an updated account of the incident indicated that two police officers were involved in the fatal encounter with Mr. Crutcher. Officer Tyler Turnbough used a stun gun on Mr. Crutcher, then another officer, Betty Shelby, fired the fatal shot that killed Mr. Crutcher. Both were White police officers (Tulsa World, 2019).

## APPENDIX A

Items for the Identification with the Victim, Personal Threat, and Collective Action Scales

Identification with the Victim

1. I feel connected with Mr. Crutcher.
2. I identify with Mr. Crutcher.
3. I feel similar to the friends and family of Mr. Crutcher.
4. I could put myself in the shoes of Mr. Crutcher.

Personal Threat

1. I feel personally threatened by the police officers' shooting of Mr. Crutcher.
2. I feel like what happened to Mr. Crutcher could happen to me.
3. I feel at risk due to the police officers' shooting of Mr. Crutcher.
4. I personally feel vulnerable because of the police officers' shooting of Mr. Crutcher.

Collective Action

1. I would participate in collective action to prevent things like the police officers' shooting of Mr. Crutcher from happening again.
2. I would sign a petition to prevent things like the police officers' shooting of Mr. Crutcher from happening again.
3. I would like to participate in a march or rally to keep things like the police officers' shooting of Mr. Crutcher from happening again.
4. I would like to join an activist group to prevent things like the police officers' shooting of Mr. Crutcher from happening again.
5. I would do something with other people to stop things like the police officers' shooting of Mr. Crutcher from happening again.
6. I would donate to charities such as the Institute for Community Police Relations or the NAACP Legal Defense Fund to help prevent things like the police officers' shooting of Mr. Crutcher from happening again.

Note: Ratings for each statement were made on a 5‐point Likert scale from “Strongly disagree” to “Strongly agree.”
